# The Diagnosis of Organic Heart Trouble

**Published:** 1886-11

**Authors:** Emory Lanphear

**Affiliations:** Kansas City, Mo.


### *THE DIAGNOSIS OF ORGANIC HEART TROUBLES.*

By EMORY LANPHEAR, Kansas City, Mo.

There are no problems of physical diagnosis which so puzzle the average practitioner as differentiating between, and recognizing the significance of, the murmurs present in organic diseases of the heart.

It is quite evident that proper therapeutic agents cannot be employed until an exact knowledge of the conditions present in any particular case can be obtained by the attending physician. In most cardiac affections attended by organic change there are distinct murmurs discoverable, and it is only by a proper understanding of these morbid sounds that an accurate diagnosis can be made. Therefore, any guide to their meaning must be acceptable to the majority of the medical profession. To those who hear, but fail to appreciate the precise meaning of these sounds, the subjoined table will prove invaluable.

I am indebted to my friend, Prof. A. B. Shaw, of St. Louis, for this table, he having presented it to the class at the Missouri Medical College in the spring of 1879. Many complex tables have been given to the profession, but this is probably the best, combining, as it does, simplicity with easiness of remembrance, yet comprising all that is needed in making a stethescopic examination of the heart, stating perfectly the time and location of the murmur, thus indicating what the lesion is, and where it is located. I submit it to the readers of this article, trusting it may prove of as much benefit to them as it has to me:

TABLE OF CARDIAC MURMURS.

| Where Heard. | Time of Murmur. | Significance. |
|---|---|---|
| Apex. | Systolic. | Mitral Regurgitation. |
| | Pre-Systolic. | Mitral Obstruction or Direct Mitral. |
| Base of Heart and Ascending Aorta. | Systolic. | Aortic Obstruction or Direct Aortic. |
| | Diastolic. | Aortic Regurgitation. |
| Base of Heart, conducted toward Ensiform Cartilage. | Diastolic. | Aortic Regurgitation. |
| Base, conjoined with Jugular Pulsation. | Systolic. | Tricuspid Regurgitation. |
| Region of Pulmonary Artery. | Systolic. | Pulmonary Obstruction. |
| | Diastolic. | Pulmonary Regurgitation. |

Pages might be written explanatory of this table; in fact, it covers the whole subject of the diagnosis of organic diseases of the heart. With it, all that is necessary is a knowledge of the topographical anatomy of the præcordial region; the location of various structures mentioned being known, and the several murmurs being heard, all that remains is to distinguish between systolic and diastolic sounds, and the diagnosis is accomplished. Without some such table in one's mind, it is impossible to intelligently examine a chest for cardiac trouble.—*Kansas City Medical Index.*

